# Supplementary figures and images for: The AMD-associated genetic polymorphism CFH Y402H confers vulnerability to Hydroquinone-induced stress in iPSC-RPE cells
Source: Front Immunol. 2025 Feb 6;16:1527018. doi: 10.3389/fimmu.2025.1527018 (PMC11839594; doi:10.3389/fimmu.2025.1527018)

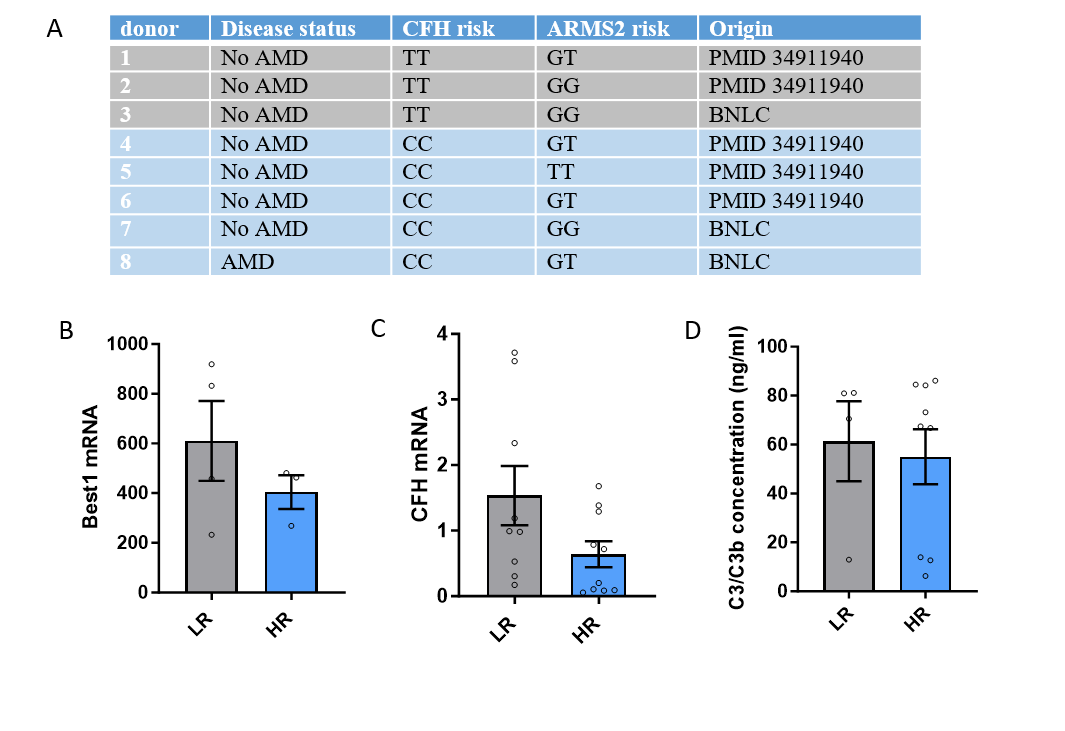

Supplement: Supplementary Figure 1 — Generation of iPSC-RPE cells (A) Summary table with the information relative to genetics and origin of the iPSC lines. Spanish National Stem Cell Bank (BNLC). (B) Gene expression levels of RPE marker BEST1 analyzed via RT-qPCR in LR and HR iPSC-RPE. Data are normalized to Best1 expression in hTERT-RPE1 cells. (C) Gene expression levels of CFH analyzed via RT-qPCR in LR and HR iPSC-RPE. (D) C3/C3b levels analyzed by ELISA in cell culture supernatants in LR and HR iPSC-RPE. Data are shown as mean ± SEM. [file Image1.tif]

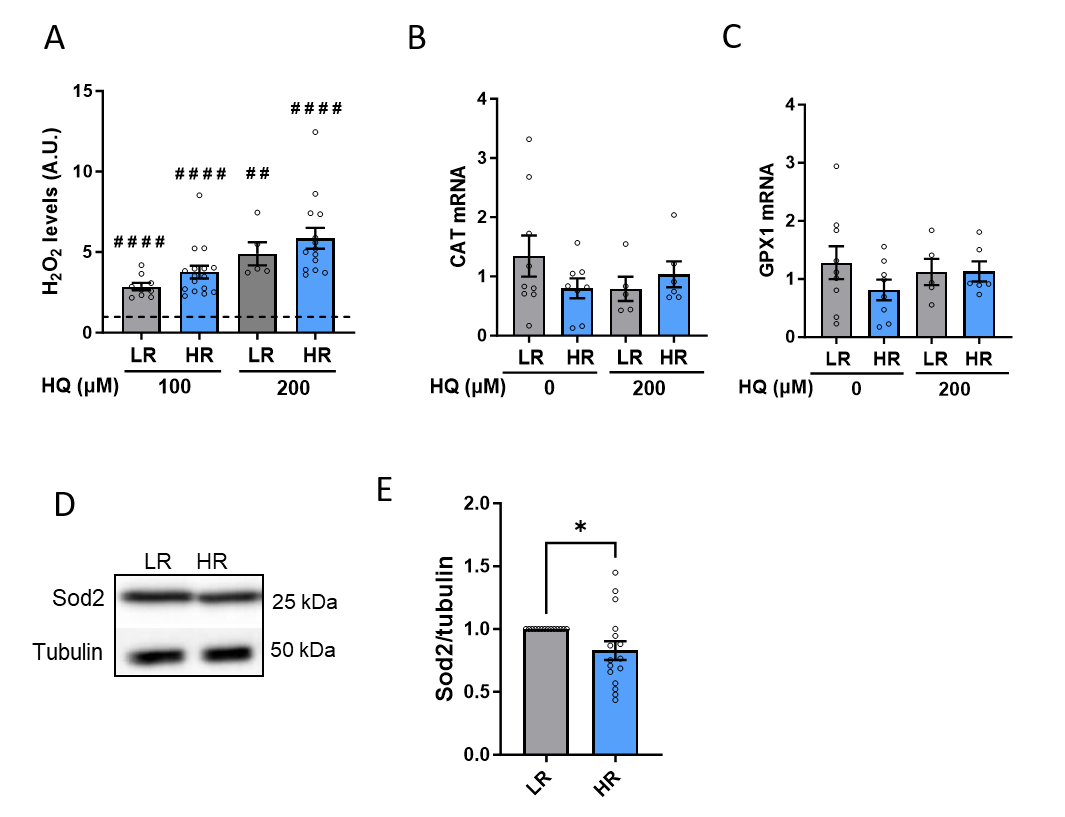

Supplement: Supplementary Figure 2 — Hydroquinone (HQ) impact on oxidative stress in LR and HR iPSC-RPE. (A) H2O2 levels assay in LR and HR iPSC-RPE. HQ-treated relative values are normalized to the respective controls (dotted line) in each individual experiment for each cell line. HQ effects in each group were assessed with paired Student’s t-test (#) compared to controls (dotted line). (B, C) Gene expression levels of CAT (B) and GPX1 (C) analyzed via RT-qPCR in LR and HR iPSC-RPE treated with HQ. (D, E) Representative WB images (D) of Sod2 levels LR and HR iPSC-RPE. Tubulin was used as housekeeping control. Quantification of Sod2 levels is shown in E. Differences between LR and HR groups were determined with one-way ANOVA (*). Data points were collected from LR iPSC-RPE1, 2, 3 (n=3 biological replicates) and from HR iPSC-RPE4, 5, 6, 7, 8 (n=5 biological replicates). Data are shown as mean ± SEM. [file Image2.tif]

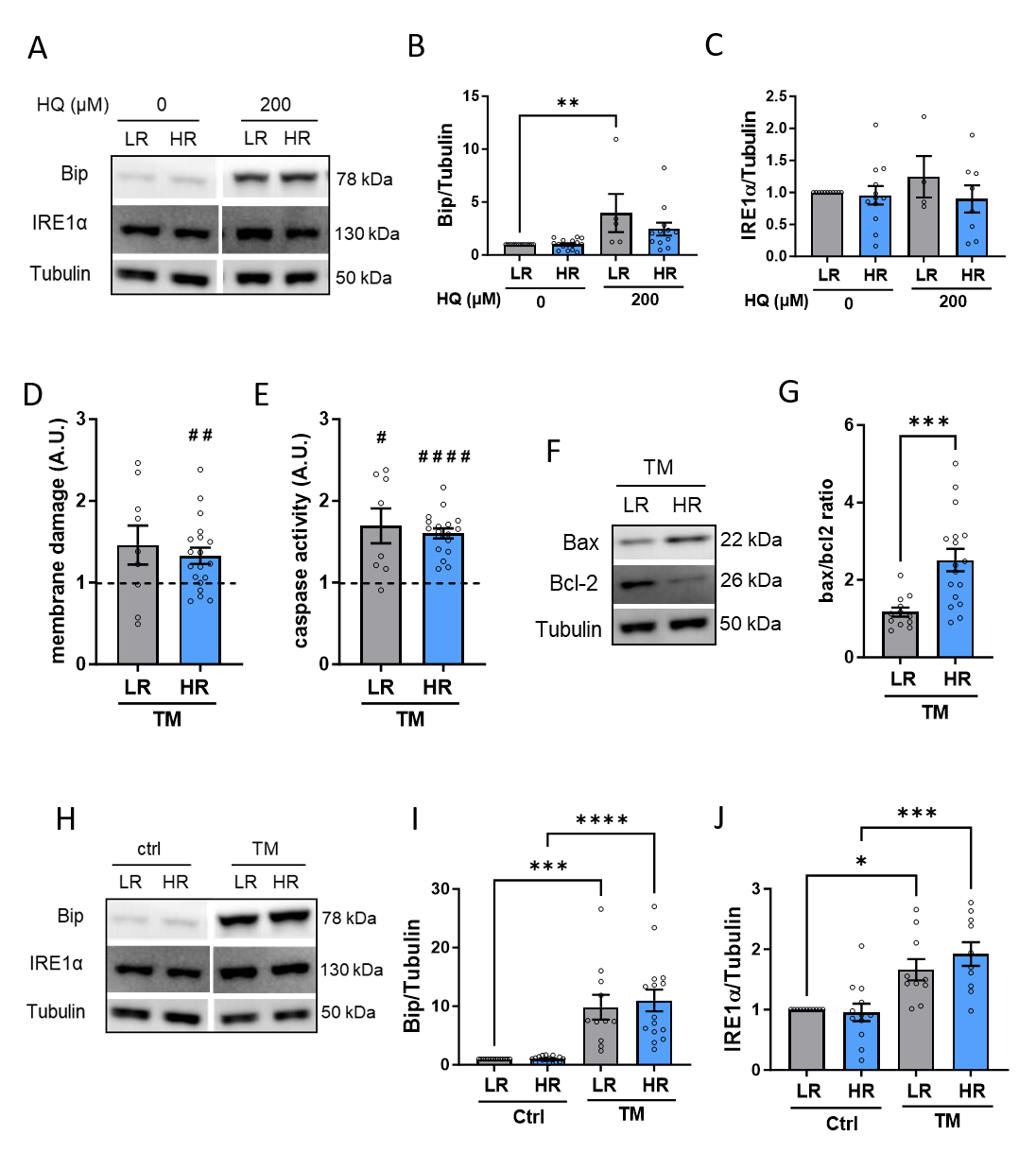

Supplement: Supplementary Figure 3 — Impact of HQ and TM on the UPR response of LR and HR iPSC-RPE. (A–C) Representative WB images (A) of Bip and IRE1α levels in LR and HR iPSC-RPE cells treated with HQ. Tubulin was used as housekeeping control. Quantification of Bip (B) and IRE1α (C) levels are shown. Differences between LR and HR groups were assessed with one-way ANOVA (*). Data are shown as mean ± SEM. (D, E) Membrane damage assessed by cytotoxicity assay GF-AFC (D) and caspase3 activity (E). TM-treated relative values are normalized to the respective controls (dotted line) in each individual experiment for each cell line. TM effects in each group were determined with paired Student’s t-test (#) compared to controls (dotted line). (H–J) Representative WB images (H) of Bip and IRE1α levels in LR and HR iPSC-RPE cells treated with TM. Tubulin was used as housekeeping control. Quantification of Bip (I) and IRE1α (J) levels are shown. Differences between LR and HR groups were assessed with one-way ANOVA (*). Data are shown as mean ± SEM. Data points were collected from LR iPSC-RPE1, 2, 3 (n=3 biological replicates) and from HR iPSC-RPE4, 5, 6, 7, 8 (n=5 biological replicates). [file Image3.tif]
